# Implementation of a Multidisciplinary Guideline to Reduce Post-operative Acute Kidney Injury in Elective Surgical Patients: A Quality Improvement Study

**DOI:** 10.7759/cureus.102842

**Published:** 2026-02-02

**Authors:** Joseph Sciacca, Stephen Pearlman, Marcin Jankowski, Brittany Anderson, Kajal Bhatia, Richard J Caplan, Jennifer Brettler, Matthew Powell, Ray Blackwell, Asanthi Ratnasekera

**Affiliations:** 1 Surgery, ChristianaCare Health System, Newark, USA; 2 Clinical Effectiveness, ChristianaCare Health System, Newark, USA; 3 Institute for Research on Equity and Community Health (iREACH), ChristianaCare Health System, Newark, USA; 4 Clinical Documentation and Integrity, ChristianaCare Health System, Newark, USA; 5 Anesthesiology, ChristianaCare Health System, Newark, USA

**Keywords:** elective, guideline, implementation, post-operative aki, surgical

## Abstract

Introduction

Post-operative acute kidney injury (AKI) has a significant impact on patients and healthcare systems. We studied the impact of the implementation of a comprehensive multidisciplinary guideline and risk assessment tool on the need for dialysis and hospital length of stay (LOS) in patients undergoing elective surgery.

Methods

We performed a single-center retrospective analysis of patients undergoing elective surgery before and after the implementation of a comprehensive multidisciplinary guideline for decreasing post-operative AKI from January 2021 to August 2024. The guideline utilized a pre-operative risk assessment tool to predict patients at high risk for post-operative AKI and utilized intra-operative and perioperative interventions to improve perfusion and volume status. Patients were categorized into pre (PRE) - January 2021 to May 2021, and post-implementation (POST) - June 2023 to August 2024, cohorts. The primary outcome was the need for dialysis. Secondary outcomes were mortality, hospital LOS, ICU LOS, and ventilator days.

Results

Of the 11,006 patient visits, 7,398 (67%) were in the PRE period, and 3,608 (33%) were in the POST period. The POST group was older (age in years POST: 63.9 vs. PRE: 62.9) and had a lower incidence of CAD (POST: 21.2% vs. PRE: 25.9%) and hypertension (POST: 47.5% vs. PRE: 52.8%). After matching, the POST group had longer procedure times and higher intra-operative fluids, and the distribution of surgical specialties was different. The POST had lower mortality (0.5% vs 1.0%, p=0.04) and shorter LOS in the hospital (median: three vs four days), in the ICU (eight vs nine days), and on the ventilator (one vs two days). In the multivariable regression analysis, the POST cohort was significantly associated with a reduction in AKI incidence (OR: 0.83, 95% CI: 0.69, 0.99; p = 0.04).

Conclusion

Implementing a multidisciplinary process to improve post-operative AKI may improve AKI and hospital LOS. Targeted interventions to reduce AKI should be further examined.

## Introduction

Acute kidney injury (AKI) is a multifactorial condition that refers to the abrupt decrease in kidney function, leading to a retention of nitrogenous waste and eventual dysregulation of fluid volumes and electrolytes. In reported studies, the incidence of AKI is variable based on country of origin, study population, and comorbidities [1,2]. However, a meta-analysis found a worldwide incidence of AKI of 21.6% in adults [2]. A higher incidence was noted in critically ill and cardiac surgery patients. A single institution study of post-surgical patients demonstrated a 32% incidence of AKI with associated long-term risk of mortality [3]. Development of AKI is known to increase morbidity and the utilization of patient care resources, accounting for 20% of intensive care unit (ICU) admissions, increased hospital cost, length of stay (LOS), and mortality [4]. Unfortunately, the rate of AKI requiring dialysis has continued to rise by 230% between the years 2000 and 2014, with diabetes, hypertension, and advanced age as key risk factors for its development [5,6]. Perioperative AKI has a significant impact on individuals and the healthcare systems. Despite this, the incidence of AKI in elective non-cardiac surgical patients is not well defined. Reported studies have variable populations, including emergency, non-cardiac, and cardiac patients [4,7,8].

Our local institution's observed-to-expected (O/E) ratios of AKI in 2021 were significantly higher when compared to other similar academic medical centers in the Vizient database. Process improvement review revealed significant variation in the approach for the prevention of post-operative AKI in elective surgical patients. Given the importance of primary prevention of post-operative AKI, our goal was to develop and implement a multidisciplinary comprehensive protocol to decrease the incidence of AKI in patients undergoing elective general surgery, colorectal, vascular, thoracic, and neurosurgical procedures. We aimed to examine whether the implementation of this guideline was associated with a decreased incidence of post-operative AKI and requirement for dialysis.

## Materials and methods

Study design and setting

Our institution is a tertiary care academic medical center with a large catchment throughout urban and suburban settings. We conducted a retrospective analysis of patients undergoing elective surgery before and after the implementation of a comprehensive multidisciplinary protocol for decreasing post-operative AKI from January 2021 to August 2024. Institutional Review Board approval was obtained and was deemed exempt as a quality improvement project. A multidisciplinary team of physicians from surgery, anesthesia, medicine, nephrology, clinical documentation and integrity, as well as data scientists, patient safety experts, and nursing leaders, was convened in November 2022. The team reviewed our existing data on post-operative AKI and specific clinical cases for opportunities for improvement. The team reviewed existing literature and performed a gap analysis. Through an iterative process, a new clinical practice protocol for the prevention of post-operative AKI was developed in April 2023 and implemented throughout the organization in June 2023. This study is being reported in accordance with the SQUIRE 2.0 Guidelines [9].

The guideline

The protocol utilized the Postop-MAKE tool, a validated risk assessment tool that predicts patients at high risk for developing post-operative AKI based on pre-operative risk factors [10]. The 11 components assessed with the tool include age, history of congestive heart failure, history of diabetes mellitus (DM), hypertension (HTN) requiring medication, emergency surgery, sepsis before surgery, preoperative creatinine, pre-operative hematocrit, serum sodium, and the type of surgery. The predictive tool provides a percent likelihood of developing a (1) acute kidney injury and (2) requiring hemodialysis, in the 30 days following surgery [10].

Following this predictive risk assessment, patients were considered "high risk" if their risk of developing AKI was greater than 2%. These patients were further evaluated by nephrology or medical providers in our pre-operative optimization clinic. Optimization may have included assessment and adjustments of diuretic and antihypertensive medications, the need for pre-operative cardiac evaluation, and guidance on pre-operative fluid intake. Intra-operatively, patients were closely monitored for hypotension using non-invasive continuous blood pressure monitoring utilizing the FloTrac® system (Edwards Lifesciences Corp, Irvine, CA). Episodes of intra-operative hypotension were defined as a mean arterial pressure less than 65 mmHg for greater than 15 minutes and were treated with vasopressors, crystalloids, or blood products based on the clinical scenario. Our protocol recommended the utilization of lactated Ringer's solution as the primary resuscitative crystalloid. In cases with high estimated blood loss, fluid resuscitation was performed with blood products. The resuscitative goal was for euvolemia as determined by the anesthesia clinician based on mean arterial pressure, stroke volume, and cardiac output goals. In addition, the protocol emphasizes avoiding hypothermia (less than 35.0 °C), adhering to standard perioperative glycemic control, and avoiding specifically identified nephrotoxic medications perioperatively. The post-operative phase of our protocol focused on the disposition of the patient who was deemed high risk, assessing the appropriate hospital unit that would best meet the patient's needs. Post-operative disposition was based upon a multidisciplinary discussion between the surgical and anesthesiology teams, who assessed the patient's pre-operative risk, intra-operative concerns or complications, and potential need for dialysis. Certain aspects of the protocol were implemented for those not identified as high risk, such as hemodynamic, temperature, and glycemic management. Goal-directed fluid resuscitation utilizing the FloTrac device was not utilized in the population who were not identified as high risk by the Postop-MAKE tool. An education plan was developed for the dissemination of the new protocol. To maximize provider awareness and adherence to the policy, the protocol was presented at multidisciplinary grand rounds, monthly scheduled meetings to surgeons, residents, medical specialists, anesthesiologists, and associated perioperative staff. Following implementation of the protocol, cases of patients who developed renal impairment following elective surgery were reviewed for process improvement, and feedback was given to the clinicians involved in their care. The team followed Vizient data and created an AKI dashboard to track monthly incidents of AKI and patients requiring dialysis. The AKI process improvement committee continues to meet monthly to review data, address concerns, and identify opportunities for improvement.

Data collection

We retrospectively collected the following variables for patients undergoing elective surgical procedures: type of surgery, age, race, sex, pre-operative creatinine, maximum post-operative creatinine, intra-operative fluid volume, and comorbidities such as chronic kidney disease (CKD), coronary artery disease (CAD), HTN, DM, chronic obstructive pulmonary disease (COPD). The elective surgeries performed in the study years are listed in a supplementary table (Appendix). Patients who did not have elective surgery; those who had cardiac, transplant, or urologic surgery; patients presenting with end-stage renal disease requiring dialysis; those who presented for dialysis access surgery were excluded from the study. Post-operative AKI was defined as an increase in baseline creatinine more than 1.5 times within seven post-operative days or an increase of baseline creatinine level by 0.3 mg/dL within 48 hours post-operatively. Patients were followed through discharge from the hospital.

Statistical analysis

Patients in the post-implementation (POST) period were matched to patients in the pre-implementation (PRE) period using propensity scores with nearest neighbor matching. Patient characteristics are summarized, showing both the total study population and the matched population. Chi-squared tests were used in matched group intergroup comparisons of categorical variables, and categorical variables are expressed as numbers and percentages. Non-parametric Wilcoxon rank sum tests were used in comparisons for age, hospital LOS, ICU LOS, and ventilator days. Mean ± standard deviation or median (interquartile range) are reported to measure the central tendency and variability. Multivariable logistic regression was conducted to analyze the relationship between AKI incidence and the implementation with adjustment of demographic factors (age and sex) and comorbidities (CKD, HTN, CAD, DM, COPD). G-estimation was used to give the average marginal results, which are summarized as the odds ratio of POST:PRE, with 95% confidence interval and p-value. Tests are two-sided. P-values less than 0.05 without adjustment for multiple comparisons are considered statistically significant. The calculations were performed using R statistical software (v4.5.1; R Development Core Team, Vienna, Austria).

Cost analysis

A cost analysis was conducted using the institutional software application (Alliance Decision Support) by Harris Affinity. Data were obtained from the patient billing system, financial accounting system, and ad-hoc statistics, which included physical areas, full-time equivalents, meals, etc. Year-to-date cost rates are calculated monthly, resulting in “cost result by cost component” rates at the charge code level. These cost rates are then assigned to encounters at the charge code level. All the steps in the costing process are reconciled to our financial statements. Direct healthcare costs are calculated by identifying patient-specific activities, such as labor, medical supplies, diagnostic services, equipment usage, etc. Costs are calculated using actual utilization data and unit prices obtained from hospital financial records, payroll systems, and procurement databases.

## Results

Of the 11,006 patients, 7,398 (67%) were in the PRE period, and 3,608 (33%) were in the POST period. Demographic data, comorbidities, and clinical outcomes can be found in Table 1. The patients in the study population in the POST period were older than those during the PRE period (mean: 63.9 vs. 62.9 yrs), had a lower incidence of CAD (21.2% vs. 25.9%), and a lower incidence of HTN (47.5% vs. 52.8%), matching these characteristics. After matching, the POST group had longer procedure times and higher intra-operative fluids, and the distribution of surgical specialties was different.

**Table 1:** Demographics and Clinical Characteristics of Elective Surgical Patients at Visits in the PRE and POST Periods Categorical variables are summarized with n and % values; PRE and POST groups are compared with chi-squared tests. Continuous variables are summarized with median (Q1, Q3) values: PRE and POST groups are compared with Wilcoxon rank sum tests. The test statistics that are shown are chi-squared for categorical variables and Z statistic for continuous variables.

Variable	All PRE (N=7398)	All POST (N=3608)	Matched PRE (N=3230)	Matched POST (N=3230)	Test statistic^a^	p value
Age, Mean (SD)	62.9 (14.756)	63.9 (14.399)	64.4 (14.338)	64.4 (13.776)	0.278	0.781
Sex, n (%) Female	3483 (51.6%)	1768 (54.4%)	1773 (54.9%)	1756 (54.4%)	0.18	0.671
Race, n (%)					1.127	0.569
Black	1367 (20.3%)	645 (19.9%)	612 (19.0%)	643 (20.0%)		
Other	336 (5.0%)	148 (4.6%)	155 (4.8%)	148 (4.6%)		
White	5038 (74.7%)	2442 (75.5%)	2456 (76.2%)	2424 (75.4%)		
CKD, n (%)	911 (12.3%)	483 (13.4%)	423 (13.1%)	442 (13.7%)	0.482	0.488
CAD, n (%)	1917 (25.9%)	764 (21.2%)	681 (21.1%)	704 (21.8%)	0.486	0.486
Hypertension, n (%)	3905 (52.8%)	1713 (47.5%)	1585 (49.1%)	1559 (48.3%)	0.419	0.518
Diabetes, n (%)	2134 (28.8%)	979 (27.1%)	852 (26.4%)	884 (27.4%)	0.807	0.369
COPD, n (%)	543 (7.3%)	280 (7.8%)	263 (8.1%)	263 (8.1%)	0	1
Procedure time, Median (Q1, Q3)	105.0(60.0, 207.0)	106.0 (66.0, 184.0)	98.0 (58.0, 185.0)	107.0 (67.0, 182.0)	-3.247	0.001
Surgical specialty, n (%)					17.373	0.004
Colorectal Surgery	396 (5.4%)	226 (6.3%)	193 (6.0%)	210 (6.5%)		
General	3042 (41.1%)	1264 (35.0%)	1217 (37.7%)	1117 (34.6%)		
Neurosurgery	975 (13.2%)	539 (14.9%)	437 (13.5%)	472 (14.6%)		
Orthopedic	2313 (31.3%)	1298 (36.0%)	1091 (33.8%)	1180 (36.5%)		
Thoracic	299 (4.0%)	160 (4.4%)	141 (4.4%)	144 (4.5%)		
Vascular	373 (5.0%)	121 (3.4%)	151 (4.7%)	107 (3.3%)		
Pre-op creatinine (mg/dL), Median (Q1, Q3)	0.86 (0.71, 1.04)	0.86 (0.71, 1.05)	0.85 (0.71, 1.03)	0.86 (0.71, 1.05)	-1.371	0.17
Max post-op creatinine (mg/dL), Median (Q1, Q3)	0.88 (0.71, 1.12)	0.86 (0.70, 1.08)	0.86 (0.71, 1.10)	0.86 (0.70, 1.08)	0.8	0.424
Vasopressor use, n (%)	4738 (64.0%)	2294 (63.6%)	2045 (63.3%)	2077 (64.3%)	0.686	0.407
Intra-op fluid (mL), Median (Q1, Q3)	1800.0 (1100.0, 3000.2)	1800.0 (1200.0, 2900.0)	1699.5(1100.0 2898.8)	1800.0 (1200.0, 2849.8)	-2.416	0.016
Transfusions (mL) FFP, Median (Q1, Q3)	199.0(196.50, 295.5)	201.0 (197.0, 397.0)	295.5 (247.8, 343.3)	201.0 (197.0, 397.0)	0	1
Platelets, Median (Q1, Q3)	288.0 (194.0, 345.0)	218.0 (195.5, 284.3)	288.0 (203.0, 289.0)	218.0 (195.5, 284.3)	0.293	0.77
RBC, Median (Q1, Q3)	340.0 (340.0, 340.0)	340.0 (340.0, 340.0)	340.0 (340.0, 340.0)	340.0 (340.0, 340.0)	0.315	0.753
Intra-op glucose (mg/dL), Median (Q1, Q3)	173 (158, 191)	176 (156.5, 200)	172 (157, 189.2)	175 (156, 200)	-0.948	0.343
Intra-op temperature (°C), Median (Q1, Q3)	35.6 (34.6, 36.2)	35.7 (34.8, 36.2)	35.6 (34.3, 36.2)	35.6 (34.8, 36.2)	-2.139	0.032
First post-op Hemoglobin (g/dL), Median (Q1, Q3)	11.1 (9.8, 12.4)	11.2 (9.8, 12.4)	11.1 (9.8, 12.4)	11.2 (9.9, 12.4)	-0.323	0.746
Post-op Hemoglobin (g/dL), Median (Q1, Q3)	11.1 (9.8, 12.4)	11.2 (9.8, 12.4)	11.1 (9.8, 12.4)	11.2 (9.9, 12.4)	-0.323	0.746
Post-op Sodium (mEq/L), Median (Q1, Q3)	138 (136,141)	138 (136, 140)	138(136,140)	138(136,140)	6.895	< 0.001
Post-op disposition, n (%)					4.581	0.205
Discharged from PACU	482 (6.5%)	274 (7.6%)	204 (6.3%)	224 (6.9%)		
Floor	4059 (54.9%)	2095 (58.1%)	1860 (57.6%)	1918 (59.4%)		
ICU	1259 (17.0%)	497 (13.8%)	469 (14.5%)	443 (13.7%)		
Stepdown	1598 (21.6%)	742 (20.6%)	697 (21.6%)	645 (20.0%)		
^a^ Wilcoxon rank sum test statistic (Z) or chi-squared test statistic						

**Figure 1:**
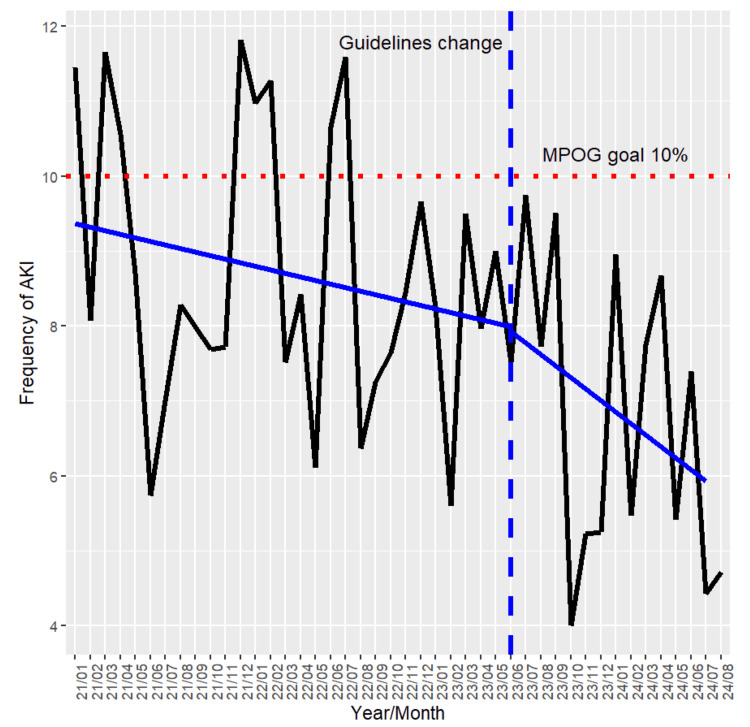
Incidences of AKI by Month in the PRE and POST Implementation Periods AKI: acute kidney injury; PRE: pre-implementation; POST: post-implementation

The POST period had lower mortality (0.5% vs 1.0%; p=0.04) and shorter length of stay in the hospital (median: three vs four days), in the ICU (eight vs nine days), and on a ventilator (one vs two days) (Table 2). The POST period did not have a significantly reduced need for dialysis (POST: 0.7% vs. PRE 0.9%; p = 0.48) or distribution of AKI stages (p = 0.24). Trends of monthly AKI incidences are demonstrated in Figure 1. The slopes were not statistically different (p = 0.41). 

**Table 2:** Primary and Secondary Outcomes in the PRE and POST Periods ^a^ Wilcoxon rank sum test statistic (Z) or chi-squared test statistic

Variable	Total (N=6460)	Matched PRE (N=3230)	Matched POST (N=3230)	Test statistic^a^	p value
Mortality, n (%)	48 (0.7%)	31 (1.0%)	17 (0.5%)	4.114	0.043
Need for dialysis, n (%)	51 (0.8%)	28 (0.9%)	23 (0.7%)	0.494	0.482
Hospital LOS (days), Median (Q1, Q3)	3.0 (1.0, 9.0)	4.0(1.0, 11.0)	3.0 (1.0, 8.0)	6.085	< 0.001
ICU LOS (days), Median (Q1, Q3)	8.0 (5.0, 14.0)	9.0 (5.0, 18.0)	8.0 (5.0, 12.0)	3.337	< 0.001
Vent (days), Median (Q1, Q3)	2.0 (1.0, 5.0)	2.0 (1.0, 6.0)	1.0 (1.0, 4.0)	4.253	< 0.001
AKI stage, n (%)				4.248	0.236
Stage 1	386 (6.0%)	207 (6.4%)	179 (5.5%)		
Stage 2	80 (1.2%)	43 (1.3%)	37 (1.1%)		
Stage 3	33 (0.5%)	20 (0.6%)	13 (0.4%)		

In the multivariable regression analysis of AKI incidence, the post-implementation period was significantly associated with a reduction in AKI incidence (OR: 0.83, 95% CI (0.69, 0.99); p = 0.04). In performing a cost analysis for the PRE cohort, the average direct cost per case was $32,564.05.

For the POST group, the average direct cost per case was $29,834.70. Thereby, a positive direct cost savings per case on average was $2,729.35. When translated to 3,805 patient visits in the POST period, the implementation of the guideline had total cost savings of $10,385,176.75 for the health system.

## Discussion

Our quality improvement study utilizing a multidisciplinary protocol to decrease post-operative AKI may have been associated with a decreased incidence of mortality and LOS in elective surgical patients. Utilization of a care bundle, including pre-operative risk assessment, assessment by nephrology and medical teams, intra-operative and post-operative goal-directed fluid resuscitation, and post-operative directed care, may be associated with reducing our primary outcome, as well as LOS, ICU LOS, and ventilator days. The risk assessment tool chosen was based on evidence review and usability. Higher levels of fluid were administered intra-operatively. There were no differences in pre-op or post-op Cr levels or AKI stages. This may be reflective of targeted and mindful volume resuscitation strategies that were utilized in the perioperative period after the implementation and education of the protocol. This may be an associated contribution by perioperative optimization, goal-directed fluid administration, and other strategies utilized. By implementation of the protocol, we also raised awareness throughout the health system of the prevalence of AKI in our elective surgical patients. We suspect that, in turn, there was a reduction of mortality, ICU, and hospital LOS, as the development of AKI has proven to be associated with prolonged LOS and death [2,11].

To our knowledge, this is the first multidisciplinary protocol-based quality improvement study demonstrating improvement of AKI needing dialysis in elective surgical patients. Our institution identified an opportunity to improve our post-operative AKI incidences based on our Vizient benchmarking data. The current literature on interventions to improve post-operative AKI remains heterogeneous. Hemodynamic management and optimal perfusion of end organs are a critical prevention strategy of AKI. The recently published randomized trial, POISE-3, examined the effect of a perioperative hypotension-avoidance strategy versus a hypertension-avoidance strategy on the risk of post-operative AKI [12]. The authors were not able to demonstrate a difference in AKI in a hypotension-avoidance strategy targeting a MAP greater than 80 mmHg in the operating room compared to a hypertension avoidance strategy. On the contrary, a systematic review published by Liu et al. demonstrated that individualized blood pressure target management significantly reduced post-operative AKI (RR: 0.67; 95% CI: 0.52-0.88) [13]. Gu et al. performed a meta-analysis of 14 cohort studies, which demonstrated that intraoperative hypotension increases the risk of post-operative 30-day mortality, cardiac events, and AKI in adult patients after non-cardiac surgery [14]. Liu et al.'s systematic review further acknowledges the heterogeneous nature of studies addressing goal-directed fluid resuscitation strategies, where patient populations, amount of fluid given, and fluid balance differ [13]. The review demonstrated that utilization of cardiac output or hemodynamic monitoring-guided resuscitation strategies did not improve AKI. Our protocol calls for maintenance of MAP goals above 65 mmHg and FloTrac-guided resuscitation in those identified as a high-risk cohort. Utilization of a bundle methodology rather than a single intervention, as proposed in our protocol, may have significance to the differing findings in our study.

Our study demonstrated an overall incidence of AKI of 8% and 1% incidence of need for dialysis in elective surgical patients, which is consistent with existing literature [7,15]. Similarly, Kork et al. performed a single-center retrospective study that examined over 39,000 non-cardiac and cardiac surgical patients utilizing the Kidney Disease: Improving Global Outcomes (KDIGO) criteria and identified post-operative AKI in 6% of surgical patients [8]. After adjusting for age, LOS, sex, and pre-operative creatinine, the study identified that a change in creatinine of 25-49% above baseline doubled the risk of death. Their findings also identified increased hospital LOS for monitoring and treatment. O’Connor et al. demonstrated an increased risk of mortality in post-operative patients who developed AKI, with an incidence of 13.3% in patients who developed AKI compared to 0.9% in those who did not [16]. The authors further identified a higher mortality at one year in patients who developed AKI compared to those who did not. These studies emphasized the significant burden of AKI for patients and health care systems.

Elghoneimy et al. demonstrated in a study of cardiac patients an increased cost of $29,000 in patients who developed AKI and progressed towards dialysis compared to those patients without renal impairment [17]. A joint consensus report of the Acute Disease Quality Initiative and Perioperative Quality Initiative, published in 2021, provided recommendations for the prevention of post-operative AKI [18]. The resource utilization for patients who develop AKI post-operatively has been extensively discussed in the literature [14-16,18]. We have demonstrated that implementation of a protocol to prevent post-operative AKI and obtaining multidisciplinary commitment from medical, nephrology, anesthesia, and surgical teams may be associated with decreased resource utilization for patients and healthcare systems. A cost savings for the healthcare system was also demonstrated with approximately $10.3 million dollars reduction in cost in the post-implementation period. Although AKI reduction consensus guidelines exist from various societies, and increased incidences of AKI, related mortality risks, and healthcare systems resource utilization are demonstrated, a comprehensive treatment protocol has not been established [19-21]. Therefore, health systems should consider the implementation of a multidisciplinary protocol to address post-operative AKI. The outcomes of studies assessing enhanced recovery pathways contributing to AKI remain heterogeneous. Drakeford et al. demonstrated that implementation of an enhanced recovery program in colorectal surgery was associated with low post-operative moderate-to-severe AKI [22,23]. However, other contrary studies did not prove a benefit of enhanced recovery pathways regarding AKI [24-26].

Our study presents several limitations. Its retrospective nature carries inherent limitations in the review of electronic health records. Further, the authors are not able to demonstrate that each patient in the post-implementation period had all the interventions recommended by the protocol. We were not able to provide any data on intraoperative hemodynamic monitoring used on identified high-risk patients. However, certain aspects of the protocol were implemented in these patients. Our patient population was heterogeneous in nature and may present a selection bias and affect the outcomes studied. However, minimizing excluded patient populations may enhance generalizability. Further, there may have been temporal changes in the institution, practice patterns, and providers caring for patients that were not accounted for in the analysis. Further, multicenter prospective studies are required to assess whether implementation of such guidelines would create a significant mortality benefit and prevent post-operative AKI. We acknowledge that implementation of a protocol may not yield significant event reduction. However, utilizing a combination of education, case review, and direct feedback to associated care providers may have an impact. Utilizing more active strategies, such as clinical decision tools in the EMR to assess risk factors and provide alerts when ordering nephrotoxic medications, may further augment implementation.

## Conclusions

Implementing a multidisciplinary process and protocol to prevent post-operative AKI may decrease incidences of AKI and thereby maximize healthcare resources. Further studies are required to examine specific interventions that would prevent AKI. Existing risk assessment tools should be inclusive of comorbidities that may be associated with AKI.

## Appendices

Supplementary Table 3 presents the list of all included procedures in the study years.

**Table 3:** List of the All Included Procedures in the Study Years

Procedure	n	%
TOTAL KNEE REPLACEMENT-TJRC	1280	0.107
CABG (CORONARY ARTERY BYPASS GRAFT)	1116	0.093
TOTAL HIP REPLACEMENT-TJRC	666	0.056
DEBRIDE/CLOSURE WOUND - EXTREMITY	470	0.039
TOTAL KNEE REVISION-TJRC	332	0.028
LAPAROSCOPIC CHOLECYSTECTOMY W/O CHOLANGIOGRAM	221	0.019
VATS4,5,6 WEDGE RESECTION/LOBECTOMY/POSS	211	0.018
LUMBAR POST LAMI W/FUSION NEURO NAV 1 L	197	0.017
LUMBAR POST LAMI W/FUSION NEURO NAV 2 L	187	0.016
CRANIOTOMY TUMOR W/IMAGE GUIDING SYSTEM W/WO CUSA	176	0.015
TOTAL HIP REVISION-TJRC	166	0.014
DEBRIDE/CLOSURE WOUND - TORSO	160	0.013
CAROTID ENDARTERECTOMY	159	0.013
AMPUTATION LEG/FOOT	158	0.013
EXPLORATORY LAPAROTOMY/POSSIBLE BOWEL RESECTION	141	0.012
LUMBAR XLIF W/POSTERIOR INSTRUMENTS 1 L	141	0.012
ROBOTIC XI SIGMOID COLON RESECTION	140	0.012
REVERSE TOTAL SHOULDER REPLACEMENT	137	0.011
AORTIC VALVE REPLACEMENT	127	0.011
AMPUTATION TOES/FINGERS	115	0.01
MITRAL VALVE REPLACEMENT	113	0.009
CERVICAL ANTERIOR DISCECTOMY FUSION 2 L	106	0.009
OH CABG/AVR (CORONARY ARTERY BYPASS GRAF	87	0.007
TAH W/WO BSO (TOTAL ABDOMINAL HYSTERECTOMY BILATERAL SALPINGO-OOPHORECTOMY)	84	0.007
CERVICAL POSTERIOR FUSION & STEALTH NAVI	81	0.007
CERVICAL ANTERIOR DISCECTOMY FUSION 1 L	80	0.007
ROBOTIC XI COLON RESECTION	80	0.007
ROBOTIC XI LOBECTOMY	77	0.006
PORTACATH/CHEMOPORT INSERTION (W/C-ARM)	70	0.006
OH AORTIC ROOT/HEMIARCH REPLACEMENT	68	0.006
LUMBAR POST LAMI W/FUSION NEURO NAV 3 L	65	0.005
URETEROSCOPY RIGID/FLEXIBLE W/LASER	65	0.005
CERVICAL ANTERIOR DISCECTOMY FUSION 3 L	64	0.005
LUMBAR POST LAMI W/FUSION NEURO NAV 4+L	64	0.005
LUMBAR POST LAMI/MICRODISC/DECOM 1 L (LA	64	0.005
LAP SLEEVE GASTRECTOMY	62	0.005
LOW ANTERIOR RESECTION	62	0.005
CYSTOSCOPY INSERTION OF STENTS W/ C-ARM	61	0.005
SMALL BOWEL RESECTION/ILEOSTOMY CLOSURE	57	0.005
TAH W/ NODES W/WO BSO (TOTAL ABDOMINAL HYSTERECTOMY BILATERAL SALPINGO-OOPHORECTOMY)	57	0.005
LAPAROSCOPIC COLON RESECTION	53	0.004
TOTAL HIP ANTERIOR REPLACEMENT MEDACTA	52	0.004
THYROIDECTOMY	49	0.004
HEPATIC RESECTION	48	0.004
ORIF ACETABULUM/PELVIS (OPEN REDUCTION I	48	0.004
ROBOTIC XI NISSEN FUNDOPLICATION/HIATAL	46	0.004
HEMICOLECTOMY, RIGHT	45	0.004
LAPAROSCOPIC GASTRIC BYPASS	44	0.004
GRAFT SPLIT THICKNESS SKIN W/DEBBRIDEMENT	43	0.004
CRANIOPLASTY	42	0.004
WHIPPLE PROCEDURE	42	0.004
FEMORAL ENDARTERECTOMY/EMBOLECTOMY	41	0.003
ABDOMINAL AORTIC ANEURYSM W/INTERAORTIC STENT	40	0.003
CERVICAL POSTERIOR LAMINECTOMY FUSION	40	0.003
LUMBAR ANTERIOR & POSTERIOR FUSION 1 L (	40	0.003
LUMBAR LAMINECTOMY W/INSITU FUSION	40	0.003
LUMBAR XLIF W/POSTERIOR INSTRUMENTS 2 L	40	0.003
COMPONENT MUSCLE SEPARATION W/ABDOMINAL	39	0.003
CRANIOTOMY TUMOR W/GLEOLAN	39	0.003
TCAR (TRANSCAROTID ARTERY REVASCULARIZAT	39	0.003
THORACIC AORTIC ANEURYSM W/ENDOVASCULAR	39	0.003
EXPLORATORY LAPAROTOMY (GYN)	38	0.003
KYPHOPLASTY	38	0.003
VP SHUNT STEALTH INSERTION	38	0.003
VATS1,2,3 WEDGE RESECTION FOR ILD/NODULE	36	0.003
CERVICAL ANTERIOR DISCECTOMY FUSION 4+ L	35	0.003
LAPAROSCOPY POSSIBLE LAPAROTOMY GEN COR	35	0.003
PARATHYROIDECTOMY	34	0.003
TOTAL SHOULDER REPLACEMENT	33	0.003
TRANSPHENOIDAL HYPOPHYSECTOMY	33	0.003
THORACIC POST LAMI W/FUSION STLTH NAV 4+	32	0.003
LAPAROSCOPIC SIGMOID COLON RESECTION	31	0.003
ROBOTIC XI HYSTERECTOMY	31	0.003
ROBOTIC XI COMPONENT SEPARATION HERNIA	30	0.003
ROBOTIC XI PROSTATECTOMY	30	0.003
TRANSVERSE LOOP COLOSTOMY	28	0.002
COLOSTOMY CLOSURE OR TAKEDOWN	26	0.002
OH VAD HEARTMATE 3 (VENTRICULAR ASSIST D	26	0.002
ROBOTIC XI LOW ANTERIOR COLON RESECTION	26	0.002
TRACHEOTOMY	26	0.002
ROBOTIC XI WEDGE RESECTION	25	0.002
SIGMOID RESECTION	25	0.002
LUMBAR POST LAMI/MICRODISC/DECOM 2 L (LA	24	0.002
ORIF TIBIAL PLATEAU	24	0.002
ROBOTIC XI ESOPHAGOGASTRECTOMY	23	0.002
ROBOTIC XI GASTRIC BYPASS	23	0.002
VENTRICULAR PERITONEAL SHUNT INSERT/REMO	23	0.002
OH MINI AVR (AORTIC VALVE REPLACEMENT)	22	0.002
CERVICAL ANT/POSTERIOR DISC FUSION 4+ L	21	0.002
ORIF ANKLE	21	0.002
ROBOTIC XI SLEEVE GASTRECTOMY	21	0.002
TUR BLADDER TUMOR (TRANSURETHRAL RESECTION)	21	0.002
COLON/SMALL BOWEL RESECTION	20	0.002
CYSTOSCOPY EVACUATION OF CLOTS	20	0.002
ORIF HUMERUS PROXIMAL (OPEN REDUCTION IN	20	0.002
ORIF METACARPALS/METATARSALS	20	0.002
CRANIOTOMY SUBDURAL HEMATOMA/INTRACEREBRAL BLEED	19	0.002
FEMORAL POPLITEAL BYPASS W/VEIN GRAFT	19	0.002
ORIF ANKLE SYNTHES (OPEN REDUCTION INTER	19	0.002
ORIF MANDIBULAR FRACTURE	19	0.002
SPINAL CORD (NEURO) STIMULATOR INSERTION	19	0.002
THORACIC POST LAMI W/FUSION STLTH NAV 1	19	0.002
GASTRECTOMY W/WO VAGOTOMY	18	0.002
IM HIP SYNTHES TFNA (TROCANTERIC FIXATIO	18	0.002
KT DONOR LAP NEPHRECTOMY (LAPAROSCOPIC)(	18	0.002
LAP VP SHUNT INSERTION (VENTRICULAR PERI	18	0.002
LUMBAR ANTERIOR & POSTERIOR FUSION 2 L (	18	0.002
MASTECTOMY MODIFIED RADICAL/SEGMENTAL/SIMPLE W/SENTINEL NODE BIOPSY/POSSIBLE AXILLARY DISSECTION	18	0.002
TEMPORAL ARTERY BIOPSY	18	0.002
CRANIOTOMY ANEURYSM/AVM	17	0.001
CRANIOTOMY TUMOR W/WO CUSA	17	0.001
DEBRIDE/CLOSURE WOUND - CRANIAL	17	0.001
FEMORAL POPLITEAL BYPASS W/GRAFT	17	0.001
GASTROJEJUNOSTOMY BYPASS	17	0.001
I&D /DEBRIDEMENT HIP (INCISION & DRAIN | 17 | 0.001 |		
IM HIP STRYKER GAMMA NAIL (INTRAMEDULLAR	17	0.001
LAPAROSCOPIC NISSEN FUNDOPLICATION	17	0.001
TOTAL HIP ANTERIOR REPLACEMENT	17	0.001
LUMBAR OLIF W/POSTERIOR INSTRUMENTS 1 L	16	0.001
LUMBAR XLIF W/POSTERIOR INSTRUMENTS 3 L	16	0.001
NECK RADICAL DISSECTION W/GLOSSECTOMY/MANDIBULECTOMY/TRACHEOTOMY/FLAP RECONSTRUCTION	16	0.001
ORIF HIP BIPOLAR/HEMIARTHROPLASTY	16	0.001
ORIF WRIST	16	0.001
REVERSE TOTAL SHOULDER ARTHROPLASTY FOR	16	0.001
CERVICAL POSTERIOR LAMI/DECOMP/TUMOR W/W	15	0.001
EXCISION LESION/MASS/CYST	15	0.001
IM FEMUR SYNTHES RFNA (RETROGRADE FIXATI	15	0.001
ORIF AC JOINT CLAVICLE	15	0.001
ROBOTIC XI CHOLECYSTECTOMY	15	0.001
ROBOTIC XI THYMECTOMY	15	0.001
STEALTH STEREOTAXIC BRAIN BIOPSY	15	0.001
THORACIC POST LAMI W/FUSION STLTH NAV 2	15	0.001
A/V FISTULA SHUNT CREATION/REVISION/DECLOTTING	14	0.001
ABDOMINAL AORTIC ANEURYSM	14	0.001
CYSTOSCOPY CHANGE STENTS;RETRO W/ C-ARM	14	0.001
I&D WOUND/ABSCESS (INCISION & DRAINAGE)	14	0.001
ORIF FEMUR	14	0.001
ORIF ZYGOMATIC/MALAR/TRIMALAR/ORBIT	14	0.001
CAROTID SUBCLAVIAN BYPASS	13	0.001
CRANIOTOMY POSTERIOR FOSSA/TUMOR W/WO CUSA	13	0.001
DEBRIDE/CLOSURE OF WOUND	13	0.001
LAP COLOSTOMY (LAPAROSCOPIC)	13	0.001
LYMPH NODE EXCISION/BIOPSY	13	0.001
MASTECTOMY SEG/NP(FAX)/SENTINEL NODE/POS	13	0.001
ORIF HUMERUS DISTAL (OPEN REDUCTION INTE	13	0.001
ROBOTIC XI PROCTOCOLECTOMY	13	0.001
SCROTAL EXPLORATION	13	0.001
BURR HOLES SUBDURAL HEMATOMA	12	0.001
CRANIOTOMY/MICROVASCULAR DECOMPRESSION	12	0.001
DEBRIDEMENT FOOT/HEEL	12	0.001
LUMBAR POST LAMI W/FACET SCREW FUSION	12	0.001
ROBOTIC XI ASSISTED PANCREATECTOMY/SPLEN	12	0.001
ANTIBIOTIC SPACER IMPLANTATION - KNEE	11	0.001
AORTIC FEMORAL BYPASS BILAT	11	0.001
CERVICAL TOTAL DISC W/ACDF (ANTERIOR CER	11	0.001
FASCIOTOMY ARM/LEG	11	0.001
GASTROSTOMY TUBE PLACEMENT OF	11	0.001
HERNIORRHAPHY VENTRAL	11	0.001
LAPAROSCOPIC APPENDECTOMY	11	0.001
OH ATRIAL MASS (MYXOMA) EXCISION	11	0.001
PERCUTANEOUS ENDOSCOPIC GASTROSCOPY	11	0.001
PERCUTANEOUS NEPHROSCOPY/OSTOLITHOTOMY	11	0.001
POSTERIOR DECOMPRESSION OF FORAMEN MAGNUM & C1-C2 FOR ARNOLD CHIARI MALFORMATION	11	0.001
ROBOTIC XI ILEOCECAL RESECTION	11	0.001
ROBOTIC XI MEDIASTINAL MASS RESECTION	11	0.001
ROBOTIC XI VENTRAL HERNIA	11	0.001
ASCENDING THORACIC ANEURYSM	10	0.001
COLECTOMY	10	0.001
CRANIOTOMY SUBOCCIPITAL/TUMOR W/WO CUSA	10	0.001
EXPLORATORY LAPAROTOMY W/INTRAPERITONEAL HYPERTHERMIA W/CHEMOTHERAPY	10	0.001
HEMORRHOIDECTOMY	10	0.001
HERNIORRHAPHY INGUINAL	10	0.001
I&D LUMBAR ABSCESS (INCISION & DRAINAGE)	10	0.001
IM TIBIA SUPRA PATELLA SYNTHES NAIL/ROD	10	0.001
LAPAROSCOPIC ADRENALECTOMY	10	0.001
LAPAROSCOPIC NEPHRECTOMY RADICAL HAND ASSISTED	10	0.001
LUMBAR ANTERIOR/POSTERIOR FUSION 1 L	10	0.001
ORIF HIP SYNTHES CANNUL/PERCUTANEOUS SCR	10	0.001
ROBOTIC XI NEPHRECTOMY PARTIAL	10	0.001
SUPRA PUBIC TUBE PLACEMENT	10	0.001
THORACIC POST LAMI W/FUSION STLTH NAV 3	10	0.001
AMPUTATION ARM/HAND	9	0.001
APPLICATION OF LARGE EXTERNAL FIXATOR	9	0.001
CERVICAL POSTERIOR LAMI/DECOMP/FORAMINOT	9	0.001
ENDOSCOPIC SINUS SURGERY W/WO NSR (NASAL SEPTAL REPAIR)	9	0.001
FEMORAL FEMORAL BYPASS	9	0.001
FEMORAL POPLITEAL BYPASS W/WO ANGIOPLAST	9	0.001
ILEO-LOOP W/CYSTECTOMY	9	0.001
IM TIBIA SYNTHES NAIL/ROD (INTRAMEDULLAR	9	0.001
LUMBAR XLIF W/POSTERIOR INSTRUMENTS 4+ L	9	0.001
MASTECTOMY BILATERAL W/WO SENTINEL NODE	9	0.001
MUSCLE BIOPSY	9	0.001
ORIF CALCANEOUS SYNTHES (OPEN REDUCTION	9	0.001
PANCREATECTOMY	9	0.001
PHACOEMULSIFICATION W/WO IOL	9	0.001
ROBOTIC XI GASTRECTOMY	9	0.001
ANTIBIOTIC SPACER IMPLANTATION - HIP	8	0.001
ILEOSTOMY	8	0.001
LAPAROSCOPY DIAGNOSTIC/OPERATIVE - GEN	8	0.001
LUMBAR POST LAMI W/ILIF 2 L (INTERLAMINA	8	0.001
NECK MASS EXCISION/DISSECTION	8	0.001
REVISION AMPUTATION LEG/FOOT	8	0.001
ROBOTIC XI INGUINAL HERNIA	8	0.001
ROBOTIC XI LAP LAP - GEN (LAPAROSCOPY/LA	8	0.001
SCREWS & PLATES REMOVAL OF	8	0.001
VERTEBROPLASTY	8	0.001
AXILLO FEMORAL BYPASS	7	0.001
CABG REDO (CORONARY ARTERY BYPASS GRAFT)	7	0.001
CERVICAL ANT/POSTERIOR DISC FUSION 3 L	7	0.001
COLOSTOMY	7	0.001
CYSTOSCOPY REMOVAL OF STENTS	7	0.001
DEBRIDE/CLOSURE WOUND - CERVICAL (ANTER	7	0.001
FEMORAL TIBIAL BYPASS	7	0.001
HEMICOLECTOMY, LEFT	7	0.001
HYSTEROSCOPY W/WO D&C /OPERATIVE (DILATION & CURETTAGE)	7	0.001
IM HUMERAL SYNTHES NAIL/ROD (INTRAMEDUL	7	0.001
LAPAROSCOPIC ASSISTED INSERTION OF GASTROSTOMY/JEJUNOSTOMY TUBE	7	0.001
LUMBAR OLIF W/POSTERIOR INSTRUMENTS 2 L	7	0.001
MORPHINE PUMP INSERTION	7	0.001
NEPHRECTOMY RADICAL	7	0.001
OPCAB (OFF PUMP CORONARY ARTERY BYPASS)	7	0.001
ORIF FEMUR DISTAL	7	0.001
ORIF FOREAR/ULNA/RADIUS	7	0.001
ORIF PATELLA	7	0.001
ORIF PILON FRACTURE (OPEN REDUCTION INTE	7	0.001
PERICARDIAL WINDOW	7	0.001
RECTOPEXY W/SIGMOID COLON RESECTION	7	0.001
SPINAL CORD STIMULATOR/BATTERY REMOVAL	7	0.001
SPINAL IMPLANT REMOVAL	7	0.001
STERNAL RE-ENTRY	7	0.001
SUPRACLAV/SCAL/NEURO/1ST RIB	7	0.001
THORACIC POSTERIOR LAMINECTOMY TUMOR W/W	7	0.001
TOTAL SHOULDER REVISION	7	0.001
ANTERIOR LUMBAR FUSION	6	0.001
ARTHRODESIS (FUSION) ANKLE W/WO GRAFT	6	0.001
AXILLARY MASS/NODE BIOPSY/EXCISION	6	0.001
BONE FLAP REPLACEMENT	6	0.001
BREAST IMPLANT/EXPANDER EXCHANGE	6	0.001
CERVICAL POSTERIOR LAMINOPLASTY	6	0.001
CHOLECYSTECTOMY W/WO CHOLANGIOGRAM	6	0.001
CYSTOSCOPY RETROGRADES W/ C-ARM	6	0.001
DEBRIDEMENT STERNAL W/FLAP CLOSURE/PLATI	6	0.001
DELORME PROCEDURE	6	0.001
DENTAL EXTRACTIONS	6	0.001
EXCISION LESION/MASS/CYST - EXTREMITY	6	0.001
FISSURE FISTULECTOMY	6	0.001
I&D PERIRECTAL ABSCESS (INCISION & DRAINAGE)	6	0.001
IM TIBIA STRYKER NAIL/ROD (INTRAMEDULLAR	6	0.001
LAP ASSISTED ERCP (LAPAROSCOPIC)	6	0.001
LUMBAR ANTERIOR/POSTERIOR FUSION 2 L	6	0.001
OH IMPELLA INSERTION	6	0.001
ORIF WRIST SYNTHES (OPEN REDUCTION INTER	6	0.001
QUADRICEPS TENDON REPAIR	6	0.001
RIB PLATING	6	0.001
ROBOTIC XI CYSTECTOMY	6	0.001
ROBOTIC XI NEPHROURETERECTOMY	6	0.001
ROBOTIC XI TAKEDOWN OF COLOSTOMY	6	0.001
ROBOTIC XI UMBILICAL HERNIA	6	0.001
SACROILIAC JOINT FUSION	6	0.001
SPLENECTOMY	6	0.001
THORACOTOMY1,2 WEDGE RESECTION	6	0.001
ABDOMINOPLASTY WITH LIPOSUCTION	5	0
BRONCHOSCOPY/ESOPHAGOSCOPY/LARYNGOSCOPY	5	0
CANCELED IN OR W/ANESTHESIA	5	0
CERVICAL ANT/POSTERIOR DISC FUSION 1 L	5	0
CERVICAL ANT/POSTERIOR DISC FUSION 2 L	5	0
COLECTOMY, TOTAL ABDOMINAL W/ILEOSTOMY	5	0
CRANIOTOMY RETRO SIGMOID APPROACH	5	0
CYSTOSCOPY BLADDER BIOPSY	5	0
CYSTOSCOPY FLEXIBLE	5	0
CYSTOSCOPY FULGURATION	5	0
LAP CHOLECYSTECTOMY INTERVAL (LAPAROSCOP	5	0
LAPAROSCOPIC COLOSTOMY TAKE DOWN	5	0
LAPAROSCOPY POSSIBLE LAPAROTOMY	5	0
LUMBAR POST LAMI/MICRODISC/DECOM 3 L (LA	5	0
NECK RADICAL DISSECTION W/FLOOR MOUTH/BASE OF TONGUE RESECTION	5	0
OH AORTIC DISSECTION REPAIR	5	0
OH TVR (TRICUSPID VALVE REPAIR/REPLACEME	5	0
ORIF FACIAL FRACTURE/CLOSED REDUCTION	5	0
ORIF PILON FRACTURE STRYKER (OPEN REDUCT	5	0
ORIF TIBIA/FIBULA SYNTHES (OPEN REDUCTIO	5	0
PSEUDO ANEURYSM REPAIR	5	0
ROBOTIC XI NEPHRECTOMY	5	0
SHOULDER HEMIARTHROPLASTY	5	0
URETEROSCOPY RIGID/FLEXIBLE	5	0
ARTHROSCOPY KNEE	4	0
C - SECTION (CESAREAN)	4	0
CYSTOSCOPY RIGID	4	0
DEEP INFERIOR EPIGASTRIC PERFORATOR (DIE	4	0
FEMORAL IM ROD AND/OR LOCKING SCREW, REMOVAL	4	0
HERNIORRHAPHY UMBILICAL	4	0
IM FEMUR STRYKER RETROGRADE NAIL/ROD (IN	4	0
IM NAIL FIBULA	4	0
LARYNGOSCOPY MICRO	4	0
LUMBAR LAMINECTOMY TUMOR W/WO CUSA	4	0
LUMBAR POST LAMI W/ILIF 1 L (INTERLAMINA	4	0
MASTECTOMY SEG/SCOUT/SENTINEL NODE/POSS	4	0
OH ECMO (EXTRACORPOREAL MEMBRANE OXYGENA	4	0
OH INSERTION PACING LEADS	4	0
OMAYA RESERVOIR INSERTION	4	0
ORIF HEEL	4	0
ORIF HUMERUS SHAFT (OPEN REDUCTION INTE	4	0
PERCUTANEOUS RADIOFREQUENCY TRIGEMINAL RHIZOTOMY	4	0
ROBOTIC XI ADRENALECTOMY	4	0
SPACIAL FRAME APPLICATION	4	0
STERNOTOMY, THYMECTOMY	4	0
TENDON/LIGAMENT REPAIR	4	0
THORACOTOMY3,4 LOBECTOMY/PNEUMONECTOMY	4	0
TOTAL HIP ANTERIOR REPLACEMENT SMITH & N	4	0
TOTAL HIP RESTORATION - STRYKER	4	0
UNICOMPARTMENTAL KNEE REPLACEMENT	4	0
VENTRICULAR PERITONEAL SHUNT EXTERNALIZA	4	0
VITRECTOMY POSTERIOR 25 GA W/LASER	4	0
BREAST RECONSTRUCT W/IMPLANT OR EXPANDER	3	0
CERVICAL LYMPH NODE BIOPSY	3	0
CERVICAL TOTAL DISC REPLACEMENT	3	0
CYSTOSCOPY BLADDER CALCULUS LASER	3	0
EGD (UPPER ENDOSCOPY) (COR)	3	0
ESOPHAGOGASTRECTOMY	3	0
EXAM UNDER ANESTHESIA	3	0
EXTERNAL FIXATOR REMOVAL	3	0
GRAFT FULL THICKNESS SKIN/EXCISION & FLAP	3	0
HICKMAN/PORTACATH/CHEMOPORT REMOVAL	3	0
ILIAC ARTERY ANEURYSM REPAIR W/ ENDO GRA	3	0
LAFORTE OSTEOTOMY/MAND/MAX	3	0
LAP ASSISTED LIVER RESECTION (LAPAROSCOP	3	0
LAPAROSCOPIC NEPHRECTOMY	3	0
LUMBAR ANTERIOR/POSTERIOR FUSION 3 L	3	0
LUMBAR OLIF W/POSTERIOR INSTRUMENTS 3 L	3	0
LUMBAR POST LAMI/MICRODISC/MIN INVASIVE	3	0
LUMBAR XLIF W/XL PLATE (EXTREME LATERAL	3	0
MANDIBULAR LESION EXCISION OF	3	0
MASTECTOMY MODIFIED RADICAL	3	0
MAXILLARY LESION EXCISION	3	0
MEDIASTINOSCOPY1	3	0
MEDIASTINOSCOPY3 POSS THORACOTOMY3	3	0
MYOMECTOMY	3	0
NECK RADICAL DISSECTION W/LARYNGECTOMY	3	0
ORIF ANKLE STRYKER (OPEN REDUCTION INTER	3	0
ORIF ELBOW SYNTHES (OPEN REDUCTION INTER	3	0
PANNICULECTOMY	3	0
PROCTO-COLECTOMY	3	0
PROCTO-COLECTOMY W/ILEO-ANAL	3	0
RETROPERITONEAL MASS EXCISION	3	0
ROBOTIC XI SACRAL RECTOPEXY W/SIGMOID RE	3	0
SACROSPINUS LIGAMENT SUSPENSION, PARAVAG	3	0
SPINAL IMPLANT REMOVAL CERVICAL	3	0
STERNAL DEBRIDEMENT WITH FLAP CLOSURE	3	0
SUBMAX/SUBMAND GLAND/MASS EXC	3	0
THORACIC POST LAMI/MICRODISC/DECOM 1 L (	3	0
VAGINAL HYSTERECTOMY, UTEROSACRAL LIGAME	3	0
ABDOMINAL PERINEAL RESECTION	2	0
ADRENALECTOMY	2	0
ARTHROSCOPIC BANKART REPAIR OF SHOULDER	2	0
AXILLARY BRACHIAL ARTERY BYPASS	2	0
BRACHIAL EMBOLECTOMY	2	0
BREAST REVISION/RECONSTRUCTION	2	0
BRONCHOSCOPY W/STENT PLACEMENT	2	0
CLOSED REDUCTION FRACTURE	2	0
CLOSED REDUCTION MANDIBULAR FRACTURE	2	0
CRANIOTOMY COLLOID CYST W/ENDOSCOPIC RES	2	0
DEBRIDE/CLOSURE WOUND - DIGIT	2	0
FEMORAL POPLITEAL BYPASS IN SITU	2	0
FREE FLAP SALVAGE	2	0
HARTMANN REVERSAL	2	0
HEMATOMA EVACUATION	2	0
HERNIA HIATAL TRANSTHORACIC/BELSY	2	0
HERNIORRHAPHY INCISIONAL	2	0
IM FEMUR STRYKER RECON NAIL/ROD (INTRAME	2	0
IM TIBIA PRE PATELLAR STRYKER NAIL/ROD (	2	0
JEJUNOSTOMY	2	0
LAP TOTAL HYSTERECTOMY (LAPAROSCOPIC)	2	0
LAPAROSCOPIC TRANS-ABDOMINAL INGUINAL HERNIA REPAIR	2	0
LIPOSUCTION MIN	2	0
LUMBAR LAMINECTOMY FOR TUMOR W/WO FUSION	2	0
LUMBAR OLIF W/POSTERIOR INSTRUMENTS 4 L	2	0
MASTECTOMY SIMPLE	2	0
MINIMALLY INVASIVE ESOPHAGOGASTRECTOMY	2	0
NECK RADICAL DISSECTION FUNCTIONAL TRACHEOTOMY	2	0
ODONTOID FRACTURE / C1-C2 FRACTURE	2	0
OH VAD HEARTMATE 2 (VENTRICULAR ASSIST D	2	0
ORCHIECTOMY	2	0
ORIF FEMORAL NECK SYNTHES | 2 | 0.000 |		
ORIF TIBIA/FIBULA	2	0
PERITONEAL CATHETER REMOVAL	2	0
PROSTATE BIOPSY MAJOR	2	0
RESTORATIVE DENTISTRY	2	0
ROBOTIC XI HEPATIC RESECTION	2	0
ROBOTIC XI PANCREATECTOMY	2	0
ROBOTIC XI SACRAL RECTOPEXY W/WO SIGMOID	2	0
ROBOTIC XI TRANS-ORAL W/NECK DISSECTION	2	0
SUBSTERNAL THYROIDECTOMY POSSIBLE STERNAL SPLIT	2	0
THORACIC POST LAMI/MICRODISC/DECOM 2 L (	2	0
TONSILLECTOMY W/WO ADENOIDECTOMY	2	0
TOTAL VAGINAL HYSTERECTOMY W/ ROBOTIC XI	2	0
TRANS ANAL RESECT RECTAL TUMOR	2	0
TUR BLADDER NECK (TRANSURETHRAL RESECTION)	2	0
TUR PROSTATE (TRANSURETHRAL RESECTION)	2	0
ULNAR NERVE RELEASE	2	0
URETERAL REIMPLANTATION	2	0
URINARY SPHINCTER EXPLANT	2	0
VENTRICULAR PERITONEAL SHUNT REVISION	2	0
VULVECTOMY RADICAL NODE DISSECTION	2	0
WIDE EXCISION MELANOMA W/SENTINEL NODE B	2	0
ACHILLES REPAIR	1	0
ANTERIOR POSTERIOR COLPORRHAPHY	1	0
ANTIBIOTIC SPACER IMPLANTATION - SHOULDE	1	0
ARTHROPLASTY FOOT	1	0
ARTHROPLASTY TMJ	1	0
ARTHROSCOPY KNEE PCL RECONSTRUCTION (POS	1	0
ARTHROSCOPY SHOULDER W/POSS OPEN ROTATOR CUFF	1	0
ARTHROSCOPY WRIST	1	0
AXILLARY DISSECTION	1	0
BREAST IMPLANT REMOVAL	1	0
BRONCHOSCOPY/ESOPHAGOSCOPY W/PLACEMENT OF ESOPHAGEAL STENT	1	0
BUNIONECTOMY SIMPLE	1	0
BURST FRACTURE / FUSION INSTRUMENTATION	1	0
CARPAL TUNNEL RELEASE	1	0
CHOLECYSTECTOMY POSS CBDE (COMMON BILE DUCT EXPLORATION)	1	0
CLOSED REDUCTION NASAL FRACTURE	1	0
CLOSED REDUCTION/POSSIBLE PINNING METACARPALS/METATARSALS	1	0
CONVERSION TOTAL HIP REPLACEMENT	1	0
CRANIOTOMY ACOUSTIC NEUROMA W/WO CUSA	1	0
CYSTOSCOPY BOTOX INJECTION	1	0
CYSTOSCOPY URETHRAL DILATION	1	0
DISTAL CLAVICLE EXCISION	1	0
ENDOSCOPIC SINUS W/REPAIR CSF LEAK (CERE	1	0
ENDOSCOPIC TRANSANAL EXCISION TUMOR	1	0
ENUCLEATION	1	0
ESOPHAGEAL DILATION	1	0
ESOPHAGOSCOPY W/DILATION - CARM	1	0
EXCISION HYDRADENITIS	1	0
FEMORAL THROMBECTOMY/PATCHPLASTY	1	0
GLOSSECTOMY PARTIAL	1	0
GROIN NODE DISSECTION	1	0
HERNIORRHAPHY DIAPHRAGMATIC/HIATAL	1	0
HERNIORRHAPHY SCROTAL	1	0
HoLEP (HOLMIUM LASER ENUCLEATION OF PROS	1	0
I&D PERITONSILLAR ABSCESS (INCISION & DRAINAGE)	1	0
ILIAC FEMORAL BYPASS/ENDARTERECTOMY	1	0
IM FEMUR STRYKER GTN NAIL/ROD (GREATER T	1	0
IM HIP SYNTHES RECON NAIL (INTRAMEDULLAR	1	0
INTERSTIM IMPLANT - PART 1 w/C-ARM	1	0
INTRATHECAL TRIAL CATH PLACEMENT W/WO DY	1	0
IR EVAR (ENDOVASCULAR ABDOMINAL AORTIC A	1	0
KT PAIRED LAP DONOR (LAPAROSCOPIC) (KIDN	1	0
LAFORTE COLPOCLEISIS	1	0
LAP DUODENAL SWITCH (LAPAROSCOPIC)	1	0
LAP GASTRIC BAND REMOVAL	1	0
LAP INSERT ESOPHAGEAL ANTI-REFLUX DEVICE	1	0
LAP SALPINGOOPHORECTOMY (LAPAROSCOPIC)	1	0
LAPAROSCOPIC SUPRACERVICAL HYSTERECTOMY W/BILATERAL SALPINGO-OOPHORECTOMY	1	0
LAPAROSCOPIC UMBILICAL HERNIA	1	0
LAPAROSCOPIC VENTRAL HERNIA	1	0
LAPAROSCOPY POST GASTRIC BYPASS	1	0
LATISSIMUS DORSI FLAP	1	0
LUMBAR OLIF FUSION	1	0
LUMBAR POST LAMI W/ILIF 3 L (INTERLAMINA	1	0
LUMBAR POST LAMI/MICRODISC/DECOM 4+ L (L	1	0
MAMMOPLASTY REDUCTION	1	0
MASTOPEXY W/AUGMENT/ABDOMINOPLASTY W/LIP	1	0
NASAL SEPTAL RECONSTRUCTION W/WO TURBINECTOMY	1	0
NEPHRECTOMY	1	0
NERVE REPAIR HAND/ARM	1	0
OH MINI MVR (MITRAL VALVE REPLACEMENT)	1	0
OH PERICARDIAL WINDOW	1	0
OH REMOVAL PACING LEADS	1	0
OH STERNAL WIRE REMOVAL	1	0
ORAL LESION/MASS BIOPSY	1	0
ORIF ELBOW	1	0
ORIF HIP STRYKER CANNUL/PERCUTANEOUS SCR	1	0
ORIF HIP SYNTHES DHS (OPEN REDUCTION INT	1	0
ORIF HUMERUS	1	0
ORIF NASAL FRACTURE/SEPTAL REPAIR	1	0
ORIF TALUS FRACTURE W/LIGAMENT RECONST (	1	0
PAROTIDECTOMY	1	0
PATELLAR REALIGNMENT	1	0
PELVIC EXENTERATION	1	0
PELVIC LYMPH NODE DISSECTION	1	0
PENILE PROSTHESIS INSERTION	1	0
PHACOEMULSIFICATION W/IOL AND VITRECTOMY	1	0
PHACOEMULSIFICATION W/IOL CORRECTIVE LEN	1	0
PILONIDAL CYSTECTOMY	1	0
POPLITEAL ANEURYSM RESECTION	1	0
PROSTATECTOMY SUPRAPUBIC	1	0
PVP - PHOTOSELECTIVE VAPORIZATON OF PROSTATE	1	0
RADICAL CYSTOPROSTATECTOMY W/ORTHOTROPIC RESERVOIR	1	0
ROBOTIC XI 1ST RIB RESECTION	1	0
ROBOTIC XI BLADDER DIVERTICULECTOMY - GU	1	0
ROBOTIC XI LYSIS OF ADHESIONS, POSS SMAL	1	0
ROBOTIC XI SACRAL RECTOPEXY W/O SIGMOID	1	0
ROBOTIC XI SUPRACERVICAL HYSTERECTOMY W/	1	0
SALPINGO-OOPHORECTOMY	1	0
SCREWS & PLATES (DENTAL) - REMOVAL OF	1	0
SMIT SLEEVE	1	0
SPHINCTEROTOMY	1	0
TEE (TRANSESOPHAGEAL ENDOSCOPY)	1	0
THORACIC POSTERIOR LAMINECTOMY W/INSITU	1	0
THORACOTOMY6,7 DECORTICATION/PLEURAL STR	1	0
TOTAL BILATERAL KNEE REPLACEMENT-TJRC	1	0
TYMPANOPLASTY	1	0
UPPP (UVULOPHARYNGOPALATOPLASTY)	1	0
UPPP/TONSILLECTOMY/SOMNOPLASTY (UVULOPHA	1	0
URETHRAL DIVERTICULECTOMY	1	0
VAGAL NERVE STIMULATOR INSERTION	1	0
VITRECTOMY POSTERIOR 23 GA W/LASER	1	0
VITRECTOMY POSTERIOR 25G/SUTURED IOL	1	0
ZENKERS DIVERTICULUM EXCISION W/WO ESOPHAGOSCOPY	1	0
